# Biomarkers as Key Contributors in Treating Malignant Melanoma Metastases

**DOI:** 10.1155/2012/156068

**Published:** 2011-10-31

**Authors:** Camila Ferreira de Souza, Alice Santana Morais, Miriam Galvonas Jasiulionis

**Affiliations:** Pharmacology Department, Federal University of São Paulo, 04039-032 São Paulo, SP, Brazil

## Abstract

Melanoma is a human neurocristopathy associated with developmental defects in the neural crest-derived epidermal melanocytes. At the present time, at least three hypotheses were identified that may explain melanoma aetiology, as follows: (1) a model of linear progression from differentiated melanocytes to metastatic cancer cells (2) a model involving the appearance of melanoma stem-like cells, and (3) an epigenetic progenitor model of cancer. Treating metastatic melanoma is one of the most serious challenges in the 21st century. This is justified because of a subpopulation of cells presenting a remarkable molecular heterogeneity, which is able to explain the drug resistance and the growing mortality rates worldwide. Fortunately, there are now evidences sustaining the importance of genetic, epigenetic, and metabolomic alterations as biomarkers for classification, staging, and better management of melanoma patients. To illustrate some fascinating insights in this field, the genes *BRAF* ^*V600E*^ and *CTLA4* have been recognized as bona fide targets to benefit melanoma patients. Our research attempts to carefully evaluate data from the literature in order to highlight the link between a molecular disease model and the key contribution of biomarkers in treating malignant melanoma metastases.

## 1. Introduction

Historically, the first report of malignant tumor formations in the skin from anatomic sites where nevi had previously existed was proposed by Virchow [1]. Melanotic nevi and cutaneous melanoma are defined as human neurocristopathies associated with developmental defects in the neural crest-derived epidermal melanocytes. One consequence of changes in skin melanocyte development is its malignant transformation to cutaneous melanoma [2, 3]. In the sixties, Clark and coauthors described three different clinical types of primary human skin melanomas based on histological growth patterns: superficial spreading melanoma (SSM), nodular melanoma (NM), and lentigo maligna melanoma (LMM). Any melanoma, except NM, was showed to present a biphasic growth pattern: an initial and long period of time characterized by superficial growth followed by rapid deeper invasion. Curiously, NM subtype does not appear to have a superficial growth component, and it was characterized by uniform invasiveness. Moreover, they observed five anatomic levels of invasion (Clark's levels I–V), based on extracellular matrix architecture, as follows: level I (tumor cells were above the basement membrane—*in situ* melanoma), level II (tumor cells invading the papillary dermis), level III (tumor cells invading both papillary and the upper part of reticular dermis) level IV (tumor cells showing any significant invasion of the reticular dermis, where collagen begins to be organized into bundles), and level V (tumor cells invading subcutaneous tissues—metastatic melanoma). Afterwards, Reed (1976) identified acral lentiginous melanoma (ALM) as another histopathological subtype of this disease, which occurs in glabrous (palmar, plantar, and subungual) skin [4, 5]. As a whole, higher levels of invasion were correlated with poor prognosis [4]. The main purpose of this article is to discuss relevant points of view on current advances and limitations in studying and treating malignant melanoma, emphasizing how early events driving tumorigenesis might help us to understand metastatic disease, and the key role of discovering new biomarkers in this scenario.

## 2. Malignant Melanoma Progression: From Normal Melanocytes to Metastatic Stage

Nowadays, there are three main hypotheses proposed to explain malignant melanoma origin and progression. Firstly, there is a linear invasion model proposed by Clark et al. [6], in which melanoma begins to develop gradually from differentiated precursor melanocytes. The earliest step of this neoplastic process is viewed as a focal proliferation of mature melanocytes, and the resultant lesion is known as common acquired melanocytic nevus. Afterwards, there is a stage in which a melanocytic nevus is comprised by melanocytes showing nuclear atypia and an aberrant differentiation program, and the resultant premalignant lesion is known as melanocytic dysplasia. Some dysplastic or atypic melanocytes have spreading capability within the epidermis and a distinctive profile of invasive growth confined to the papillary dermis. This primary malignant lesion is termed radial growth melanoma (RGP) and does not form metastasis. The appearance of a new population of cells with aggressive biological potential, growing in an expansive way and perpendicular direction, and invading the reticular dermis is correlated to a more dangerous step known as primary vertical growth melanoma (VGP). VGP melanoma is clinically dangerous because of a cell subpopulation presenting competence for metastasis. However, these cells and surrounding environment do not have sufficient angiogenesis or lymphangiogenesis capabilities to support metastases. VGP melanoma cells can progress to a metastatic stage, in which deeper invasion of the subcutaneous tissues and extensive neoangiogenesis are important properties. As a hallmark of metastatic developmental stage, a cluster of progeny cells are able to colonize distant tissues from the origin of the primary melanoma [6, 7]. The molecular mechanisms of invasion and metastasis require changes in cell motility gene expression, and these processes occur by coordination of cell extension, adhesion, deadhesion, and contraction steps [7]. Importantly, not all melanomas arise from melanocytes that pass through the atypical melanocytic nevus stage and not all atypical melanocytes progress to the malignant disease. The evidence in which malignant melanoma does not universally arise from nevus and that this may be the exception rather than the rule was proposed by Clark et al. in 1969 [4]. Hence, two other models were proposed. One of them is a melanoma stem cell and tumor microenvironment model, in which melanoma stem-like cells (tumor-initiating cells) lead directly to the observed clinical outcomes without progressing through intermediate stages. Additionally, fibroblasts, endothelial cells, and inflammatory cells from the tumor microenvironment would be able to contribute to and support metastases [8]. Finally, there is an epigenetic progenitor model to explain the common basis of cancer proposed by Feinberg et al. [9], in which early epigenetic changes occur in tissue-specific (noncancerous) stem-cells. According to this polyclonal model, this primary alteration can be due to events within the stem cells themselves, the influence of the stromal compartment, or microenvironmental injury. Later, epigenetic plasticity and genetic alterations would drive tumor progression [9]. Thus, the epigenetic progenitor model of cancer provides new insights to understand the malignant transformation. Since global epigenetic abnormalities accumulate during cancer development, it is reasonable to hypothesize that deregulation in epigenetic mechanisms may be involved in melanoma aetiology.

## 3. Staging of Metastatic Melanoma at Diagnosis and Prognosis

Malignant melanoma is a complex disease and patients diagnosed at metastatic stage have a poor prognosis because of the remarkable molecular heterogeneity and resistance of melanoma cells to apoptotic processes and classical chemotherapy interventions [10, 11]. Metastatic melanoma cells tend to disseminate to multiple organs, including but not limited to brain, lungs, liver, and bone. Currently, there is no effective cure for advanced disease. Complete remissions after chemotherapeutic regimens rarely benefit more than 20% of patients [11]. According to the National Cancer Institute 68,130 new cases and 8,700 deaths from malignant melanoma were estimated in the United States in 2010 (available at http://www.cancer.gov/). Taken together, these data suggest that malignant melanoma represents an important public health problem in the 21st century. 

As a result of multivariate analysis of 7,972 patients with metastatic melanoma performed by American Joint Committee on Cancer (AJCC), the clinicopathologic factors that define the M-category strata to the conventional TNM (tumor, node, *metastasis*) system should be evaluated by two dominant criteria. The first one is the site(s) of distant metastases, as follows: M1a—nonvisceral (skin, subcutaneous (soft) tissue, or distant lymph nodes), M1b—lung or a combination of lung and skin or subcutaneous metastases, and M1c—the other visceral metastatic sites. The second criterion is the increased lactic dehydrogenase (LDH) serum level that is a powerful and an independent predictor of survival among patients with stage IV melanoma. In this case, patients are all categorized as M1c, regardless of the site(s) of their distant disease. These staging for metastatic melanoma have been approved by the International Union Against Cancer (UICC) TNM Committee. One-year survival rates among 7,972 stage IV patients were 62% for M1a (better prognosis), 53% for M1b (intermediate prognosis), and 33% for M1c (worst prognosis) melanomas (*P* < 0.0001). The overall prognosis of all patients with stage IV melanoma remains poor, even among those who are classified as M1a. For this reason, the Melanoma Staging Committee recommended no stage grouping for stage IV melanomas to clinical trial studies [12].

## 4. The Key Role of Biomarkers in Melanoma Classification Refinement

It is well known that malignant melanoma development correlates with genetic alterations and sun exposure since ultraviolet radiation is the major risk factor [13]. Currently, aberrant epigenetic patterns, including DNA methylation, canonical and variant histone marks, nucleosome positioning, and noncoding RNAs (specifically microRNAs), are emerging as dynamic hallmarks of earliest and later events driving melanoma genesis and progression [14–16]. To illustrate this, we recently demonstrated that nonmetastatic and metastatic murine melanoma cells treated *in vitro*, respectively, with Trichostatin A and 5-aza-2′-deoxycytidine (pharmacological inhibitors of histone deacetylases enzymes and DNA methyltransferases, resp.) showed reduction of tumor growth *in vivo *when inoculated in syngeneic mice. Moreover, we observed changes in epigenetic machinery components even in premalignant melanocytes, suggesting that epigenetic marks act as key contributors to melanocyte full malignant transformation and melanoma aggressiveness [16]. A recent study has pointed out the role of chemicals as melanoma risk factors, such as the polychlorinated biphenyls pesticides [17]. This study uncovers the existence of other risk factors associated with melanoma development, which are not yet well characterized.

The classification scheme recommended by The World Health Organization (WHO) to identify primary melanomas distinguishes four main melanoma subtypes based on histological growth patterns: SSM, LMM, NM, and ALM [4, 5, 18]. However, there are reasons to be concerned about classical classification schemes. There is a group of pathologists who recognize a subgroup designated as malignant melanoma of unclassifiable histogenetic type [18]. The meta-analysis study conducted worldwide showed that histological type of primary melanomas did not have predictive clinical outcome once systemic metastasis had developed [19]. Moreover, distinct types of melanomas had different susceptibility to ultraviolet light [20], suggesting that not all melanomas arise from areas exposed to the sun. Because of this, another classification system has been used to classify primary melanomas arising from nonglabrous skin, as follows: melanomas occurring in nonchronically or intermittently sun-damaged skin and in chronically sun-damaged skin. This classification system comprises acral melanomas and mucosal melanomas, which can be separated based on patterns of chromosomal aberrations [20]. Furthermore, it is well known that melanoma cells express multiple molecular phenotypes. This feature has been associated with the presence of a subpopulation of cells showing stem-cell properties, such as the ability to differentiate into several mesenchymal lineages, including melanocytic cells [8]. Importantly, a recent study reported the presence of a temporarily distinct subpopulation of slow-cycling melanoma cells, which seem to be an essential feature for continuous tumor growth [21]. Apart from genetic alterations, another possible explanation to melanoma cells heterogeneity is the epigenetic reprogramming as an adaptation of cells to a sustained microenvironmental stress condition [9, 16]. Taken together, these findings highlight the urgency in understanding somehow molecular variables can predict subtypes biologically more homogeneous to refine the WHO melanoma classification scheme and improve melanoma treatment. In this way, current studies have been performed to identify reliable biomarkers to predict melanoma clinical outcome. Unfortunately, most of the studies characterizing “omics” expression do not emphasize or describe with sufficient details any melanoma classification system. This scenario was able to explain, at least in part, the poor correlation between discovered biomarkers from basic research with results of clinical trials and their potential clinical exploitation. Here, we present a compilation of studies conducted according to the new hypothesis, in which identifying patient's subgroups more biologically homogeneous needs an integrated analysis involving histomorphological measurements, transcriptome, proteomic and metabolomic studies, and epidemiologic and clinical variables. Such data suggest that this evaluation scheme is more accurate than the currently classification based only on histological features.

**Study 1**

Alonso et al. [22] performed an integrated study based on the combination of tissue microarray approach with clinical and histopathological data. The aim of the study was to identify new proteins differentially involved at specific stages of melanoma as candidate biomarkers for target therapy. A total of 175 human specimens were retrospectively evaluated, including 10 nevi, 28 RGP primary melanomas, 66 VGP primary melanomas, and 71 metastases (34 skin metastases and 37 nodal metastases). As a result, this study demonstrated that each step in malignant melanoma development was characterized by the expression of a specific signature. Among the most detected alterations observed were upregulation of cyclin A, cyclin D1, CDK1 and CDK2 (cell cycle regulatory proteins), survivin (apoptosis), and active form of STAT1 (transcription factor) in RGP melanomas relative to nevi; simultaneous upregulation of Ki-67 and downregulation of p27^KIP1^ (cell cycle regulatory proteins) in VGP melanomas relative to RGP melanomas or benign lesions; upregulation of cyclin D1 and cyclin D3, and a loss of p16^INK4a^ (cell cycle regulatory proteins), and downregulation of BCL2 (apoptosis) and MUM1 (transcription factor) in metastatic melanomas relative to VGP melanomas. Although many of identified changes are stage specific, it seems that an increasing degree of expression of cyclins and CDK(s), in conjunction with a loss of CDK inhibitors, facilitate the progression to advanced clinical and histological stages. Curiously, cyclin D1 protein expression was negative in all nevi and markedly expressed in RGP melanomas and metastases, and significantly downregulated in VGP melanomas. As discussed by authors, these findings suggest a critical role for cyclin D1 in melanoma pathology, and illustrate how melanoma progression is meticulously and dynamic coordinated. Thus, to progress from nevus to metastasis, each step is distinguished by the expression of a characteristic set of molecular marks or, perhaps, by their differential levels of expression as those observed for cyclin D1. In fact, our experience with global gene expression analysis using high-density oligonucleotide microarrays in a murine melanoma progression model supports this idea, in which several kinetic profiles of gene expression from nontumorigenic melanocytes to metastatic melanoma cell lineage were observed (see [16], and data not published). Moreover, Alonso's group [22] observed a defective deregulation of apoptosis by upregulation of survivin protein expression from nevi to RGP, VGP, and metastatic melanoma, suggesting a parallel between apoptosis resistance and melanoma progression. Since 30 patients had VGP and metastatic melanomas, a simultaneous analysis in the expression of a combination of relevant molecular markers in the same patients from both stages was performed. In this way, this study also developed a predictor model for survival identifying a group of patients with shorter overall survival associated with VGP melanoma, which was related to the absence of p16^INK4a^  or the presence of BCL6 protein expression in conjunction with either high Ki-67 expression or positive p21^CIP1^  [22]. Although apparently paradoxical because of its cyclin-dependent inhibitory and tumor suppressor activities, a recent study reported that p21^CIP1^ may also act as a tumor promoting factor by inhibiting apoptosis [23].

**Study 2**

Curtin et al. [20] performed an elegant study in which they compared genome-wide alterations in the number of DNA copies, and mutational status of *BRAF* and *NRAS*, in attempt to clarify the relationship among clinical heterogeneity, histological characteristics, degree of sun exposure and susceptibility to ultraviolet light, and genomic instability. A total of 126 tumor specimens were evaluated and classified in four groups based on their location and differences in the degree of sun exposure, as follows: 36 specimens of acral melanoma, 20 specimens of mucosal melanoma, 30 specimens of melanoma arising from skin with chronic sun-induced damage, and 40 specimens of melanoma arising from skin without chronic sun-induced damage. All primary melanomas had an invasive component in which tumor cells predominated over stromal cells. As a result, they observed and discussed that there were distinct sets of genetic alterations, suggesting that melanoma develops by different mechanistic pathways in response to different selective influences. As a whole, there were marked differences in aberrant genomic regions among the groups. These differences were more pronounced between melanomas on skin that were relatively or absolutely protected from the sun (glabrous skin in acral melanomas and mucosal epithelia membranes in mucosal melanomas) and melanomas on skin exposed to the sun. Acral and mucosal melanomas had a significantly higher degree of chromosomal aberrations, but they involved different genomic regions. The commonest alterations distinguishing these two groups were gains involving the *CCND1* locus, gains and losses involving chromosomes 22 and 4q, respectively, in the group exposed chronically to the sun, while the losses involving chromosome 10q were more frequently observed in the group with no skin-induced damages. Specifically, eighty-one percent of tumors on skin without chronic sun-induced damage frequently had mutation in *BRAF* together with fewer copies of *PTEN*, or mutations in *NRAS* alone. In accordance to this finding and as reviewed by authors, *BRAF* and *NRAS* genes do not show typical ultraviolet “fingerprint” mutations. On the other hand, the majority of melanomas in the other three groups did not have mutations in *BRAF* or *NRAS* but instead had increased copy number of the downstream genes *CCND1* or *CDK4*. These findings implicate *CDK4* and *CCND1* as independent oncogenes in melanomas presenting this genetic background (absence of mutations in *BRAF* and *NRAS*). As discussed by authors, their findings are of great clinical importance because of possible prevention and potential therapeutic strategies. Hence, in melanomas in which *BRAF* or *NRAS* mutations are present, they would be expected to be responsive to therapeutic interventions targeting the RAS-RAF-ERK and PI3K pathways, such as sorafenib [20]. In parallel, a recent multicentric study reported that the treatment of metastatic melanomas carrying *BRAF^V600E^* mutations with a selective small molecule inhibitor PLX4032 resulted in complete or partial regression of disease in most of the patients [24].

**Studies 3 and 4**

Viros et al. [25] performed a study based on genotype-phenotype point of view, in which they refined a current classification scheme of distinct melanoma subtypes proposed by WHO, integrating to this existing classification model continuous variables, such as a panel of histomorphological measures (scatter of intraepidermal melanocytes, nesting of intraepidermal melanocytes, cytoplasmic pigmentation of neoplastic melanocytes, cell size, cell shape, nuclear size, and nuclear shape), and unordered categorical variables, such as anatomic site, WHO subtype, melanomas arising from skin with and without evidence of chronic sun-induced damage, acral grouping and gender, and correlated them with the mutation status of *BRAF* (exon 15, which includes codon 600) and *NRAS* (exons 1 and 2, only if no *BRAF* mutation was detected, since there is a very low cooccurrence of these aberrations). A total of 302 primary radial growth melanomas were evaluated, and areas with vertical growth were excluded, except in the case of nodular melanoma, in which the assessment was made in any small intraepidermal component adjacent to the nodular portion wherever possible. The aim of this study was improving melanoma classification by integrating genetic and morphologic features, and clinical information to provide a better understanding regarding malignant melanoma “histogenetic” origin and group melanoma in a more homogeneous biological category. As a result, the authors observed that melanomas with *BRAF* mutations showed distinct morphological features, such as increased upward migration and nest formation of intraepidermal melanocytes, thickening of the involved epidermis, and sharper demarcation to the surrounding skin, and had larger, rounder, and more pigmented tumor cells compared to melanomas without *BRAF* mutation. Moreover, among the clinical variables, they identified age <55 years as the single most predictive factor for *BRAF* mutation. When this age cutoff was applied to an independent cohort of 4,785 patients, a better prognosis associated to the development of metastases to regional nodes were observed whereas patients that, on the basis of their age, were less likely to have a *BRAF* mutant melanoma more frequently displayed satellite, in-transit metastasis, and visceral metastasis. On the other hand, *NRAS* mutation failed in distinguishing melanomas on the basis of these morphological features. Finally, authors discussing the importance of improving the classification scheme in two ways: to facilitate stratification for therapy, as well as retrospective analysis of existing clinical trials [25]. The reproducibility of this study was validated by Broekaert et al. [26] who confirmed that *BRAF* mutation define a subset of melanomas more biologically homogeneous, which metastasized more frequently to regional lymph nodes and are commonly observed in younger people. Finally, the authors discussed the heterogeneity exhibited by melanomas without *BRAF* mutations, in which a subgroup having very similar clinical and morphological characteristics as those observed in melanomas with *BRAF* mutation was observed, suggesting the possibility that they are biologically related and, perhaps, alternative genes acting immediately downstream of *BRAF*, such as *MEK1* and *MEK2* could be candidates for target therapy in this context [26].

**Study 5**

Nguyen et al. [27] hypothesized that melanoma AJCC stages could be classified by epigenetic biomarkers. The authors performed a study in which specimens from 15 primary cutaneous melanomas, 16 lymph node metastases, and 31 distant metastases were assessed to determine the significance of microRNA-29 isoform C and DNMT3A and DNMT3B expression in melanoma progression and clinical outcome [27]. Moreover, they were interested in determining their potential utility as hallmarks of CpG island methylator phenotype (CIMP), which is associated with melanoma aggressiveness through inactivation of tumor suppressor genes and tumor-related genes, and methylation of MINT (noncoding methylated-in-tumor) loci [27, 28]. As a result, they observed that the downregulation of microRNA-29c was associated with hypermethylation status of tumor-related genes and MINT loci, and inversely correlated with DNMT3A and DNMT3B expression in metastatic tumors. These findings suggest the antagonistic role of microRNA-29c in regulating DNMT3 expression, and that its potential tumor suppressor activity is lack and correlate with melanoma progression. Furthermore, expression of microRNA-29c correlated with DNMT3B expression was found significant as prognostic factor predicting overall survival in patients with lymph node metastases. Hence, the authors discussed that microRNA-29c expression may potentially provide significant information by differentiating metastatic melanoma in the way to improve adjuvant therapy for advanced disease [27].

**Study 6**

Recently, Abaffy et al. [29] exploited an emerging approach based on volatile metabolomic fingerprint, that does not alter tissue morphology and potentially permits to discover new melanoma biomarkers (metabolites of interest that change in response to melanoma development) to improve early diagnosis and positive clinical outcome for patients. As a result, the authors observed that a differential metabolic signature of melanoma does exist, and was characterized predominantly by three volatiles expressed in fresh and frozen melanomas: 4-methyl decane, dodecane, and undecane. Curiously, the first one is a methylated alkane previously listed as a biomarker of lung cancer, suggesting an increased methylation process in melanoma. Moreover, the presence of secondary metabolites of membrane lipid peroxidation, such as dodecane and undecane, indicates an oxidative stress environment [29]. These findings are in accordance to works performed by our group, in which sustained stress resulted in higher levels of reactive oxygen species in the microenvironment [30]. This feature was associated with epigenetic reprogramming of non-tumorigenic murine melanocytes, involving both DNA methylation and histone marks, evaluated at transcriptional and posttranslational molecular levels, respectively [16]. Abaffy et al. (2010) also identified pyridine as a unique volatile biomarker of nevus. Finally, they emphasized that volatile metabolomic might help in improving melanoma classification schemes and targeted therapy [29].

The deregulation of oncogenic and tumor suppressor signaling pathways are key molecular mechanisms promoting melanoma progression. The most recently candidate biomarkers discovered from human melanoma cell lines and tumor specimens are shown in Tables 1 and 2.

Taken together, these data suggest that discovering new biomarkers might represent a powerful tool in melanoma classification refinement and their potential exploitation in clinical trial designs.

## 5. Treating Metastatic Malignant Melanoma

Over the years, finding an ideal treatment for patients with advanced melanoma has been the major challenge for researchers in this area [51]. Meanwhile, the dissemination of this malignancy to new and distant tissues, with rare exceptions, is almost a sign of incurable tumor. According to American Joint Committee on Cancer (AJCC), patients with stage IV melanoma have a median survival time of only 8-9 months, with 1-year survival rates of 33%–62% [12]. This is essentially because no treatment approach has demonstrated a survival benefit, but efforts have been done. The basis of treatment for metastatic melanoma is systemic therapy which addresses the subclinical sites of metastases as well. On the other hand, locoregional treatment modalities such as surgery or radiation are usually used as palliative approaches or as adjuvant in systemic therapy to improve clinical outcomes [52]. 

### 5.1. Classical Systemic Interventions

The systemic therapies include different methods as follows.

- Cytotoxic chemotherapy: it has been used for over three decades and continues to be the standard treatment of metastatic melanoma due to dacarbazine that is the only chemotherapeutic agent approved by the Food and Drug Administration (FDA) despite its modest efficacy. The moderate antitumor activity of these chemotherapeutic agents led to attempts to combine them but the latest studies have demonstrated that higher response rates are associated with increased toxicity without any survival advantage when compared with a single-agent regimen [53];
- Immunotherapy: term used for nonspecific as well as specific immunomodulation which, in other words, means use of natural or manufactured substances to boost or restore the body's immune system so it can better recognize and fight disease [54]. Most commonly used as adjuvant therapy for melanoma following surgery but, due to the relative success of some immunotherapeutic approaches, this strategy has fuelled extensive investigation and many clinical trials are underway. Interleukin 2 (IL-2) is a cytokine immune system activator that stimulates T-cell proliferation and function besides being used as a lymphokine-activated cell killer therapy. High-dose recombinant IL-2 was approved by the FDA in 1998 for treatment due to the potential for durable complete responses in a small cohort of appropriated patients [55]. The efficiency in administration of high-dose IL-2 is associated with significant toxicity and, consequently, multiple side effects, so its use is limited to select patients whose medical care is done very carefully. Interferon alfa-2b (INF *α*-2b), naturally produced cytokine that helps to activate the immune system, is also considered an adjuvant therapy of resected high-risk melanoma, but its effectiveness is not so much better than that already observed with IL-2;
- Biochemotherapy: this kind of treatment refers to the most intensive regimens that combine polychemotherapy or single-agent with immunological agents INF-*α* and/or IL-2. The systemic biochemotherapy has promise, but results from recent trials have been mixed and inconsistent [56–58], in part because addition to exposing patient to consistently high toxicity rates, the responses are not durable and do not improve overall survival. While the utility of biochemotherapy is really controversial, in the United States, many melanoma treatment centers continue to use this therapy for melanoma [59]. Interestingly, it was recently reported novel regimen of maintenance biotherapy and induction biochemotherapy/maintenance biotherapy to extend progression-free and overall survival, but this promising regimen will be studied in a randomized clinical trial in patients with advanced metastatic melanoma [60]. In summary, until today biochemotherapy regimens cannot be regarded as standard clinical practice and should be further evaluated in clinical trials.

### 5.2. Promising Approaches in Systemic Therapies

Several attempts to upgrade existing therapies for metastatic melanoma may not have been successful in clinical studies, but have revealed remarkable advances to our understanding of this disease. This significant progress is the foundation for new opportunities of treatments. Currently, two major trends are recognized for new treatment strategies and both are involved in the development of therapies that promote an intervention at the molecular level [61]. One of them is directly related to the mechanism of immune response of patients but with a new vision of modulation while the other aims key biomarkers presented in signaling pathways, which are highly deregulated along melanoma pathogenesis. 

The greatest optimism for advances in the immunotherapy of melanoma comes from introduction of new agents and methods that block or avoid the natural regulatory mechanism, that limit the magnitude of induced T-cell response, and the tumor-related immunosuppressive mechanism [54]. Some of the novel cancer immunotherapies deserve brief comments.

- Immunoregulatory monoclonal antibodies: cytotoxic T-lymphocyte antigen (CTLA) is expressed on activated T lymphocytes and regulatory T cells. It serves as a natural breaking mechanism that returns T cells to homeostasis following an immune response, it controls the duration and intensity of this response. Monoclonal antibodies (anti-CLTA-4 mAb) that bind to CTLA-4 inhibit this negative switch and may break peripheral tolerance to self-tissues and potentiate immune response against cancer cells. Ipilimumab, a CTLA-4 blocker of fully human IgG1 monoclonal antibody class, is the most recently FDA approval for unresectable or metastatic melanoma. Clinical trials for monotherapy and the combination with other immunotherapies and vaccines have been concluded or are currently underway [62]. Because of the unusual and severe adverse effects referred to as autoimmune breakthrough events (ABEs) or immune-related adverse events (irAEs), the therapy is being approved with a risk evaluation and mitigation strategy to inform healthcare professionals about these serious risks. Despite this fact, this drug currently holds great promise for treating patients with advanced malignant melanoma. Tremelimumab is an IgG2 monoclonal antibody also directed against CTLA-4, but the benefits and effects of its use are still being investigated [63, 64]. Many other targets related to the immune system are being investigated (Table 3) but owing to complexity of immune system and presence of molecular immunological target on multiple cell types, whose effect is cell-type specific, the predominant mechanism for an individual agent's antitumor activity often cannot be determined with certainty. Definitely, combinations presenting optimal antitumor activity and the right combination for each patient will depend on the individual's tumor biology and host factors which makes it difficult to search for an appropriate therapeutic strategy [65].

(ii) Adoptive cell transfer (ACT) is a type of adoptive immunotherapy that involves *ex vivo* activation and expansion of autologous tumor-reactive T-cell populations taken from tumor-bearing host that then are reinfused back into patient. Several factors may be obstacles to the effectiveness of this immunotherapy, such as nonpersistence infused cells* in vivo *and possible reduction of infused cells by preexisting T_reg_ cell. The recent studies demonstrated that activity of adoptively transferred T cells can be improved following on nonmyeloablative lymphodepleting regimen [66]. The lymphodepleting regimen helps to eliminate T_reg_  cell as well as normal endogenous lymphocytes that compete with the transferred cells for homeostatic cytokines and thus promoting increased circulating cytokines (IL-7 and IL-15) while nonmyeloablative chemotherapy refers to the uses of moderate doses of chemotherapy, just to suppress the immune system for a brief period. This evolutionary technique to treat cancer has demonstrated significant progress, mediating objective tumor regressions in significant percentage of patients who stayed on this treatment [67]; however, it is important to mention it is not available as standard treatment for advanced melanoma. Many studies are underway, and the new investigations are focusing the transfer of T cell genetically modified to express melanoma-specific T-cell receptors [68].
(iii) Vaccine therapy is an experimental treatment that aims to stimulate immune system to recognize the antigens on cancer cell surfaces and be able to promote an active immunity targeting these malignant cells. Various strategies are currently being tested, but, to date, no vaccination procedure has shown significant efficacy in the metastatic setting. The advance in immunotherapy field, like development anti-CTLA-4 and other immunomodulatory antibodies, may come to improve the outcomes with vaccine treatment, as they may play a crucial role in maintaining an immune response initiated by a vaccine [69]. Many studies are being conducted and positive experiences recently reported (Table 4) are extremely encouraging for the generation of new research trials in this area. On the other hand, some negative results with vaccines have also been reported, especially in the adjuvant setting, indicating that multiple vaccinations can have detrimental effects, perhaps because of induction of tolerance [70, 71]. Finally, the researches are still continuing and, despite some previous results, seem promising, there are many points yet to be clarified, especially regarding the manner in which the patient's body assimilates this type of therapy, which is difficult clinical progress.

All these data lead to the conclusion that although many advances have been achieved with immunotherapy, under combined regimes and/or using the novel immunotherapic agents, this option for treatment still shows certain limitations to an appropriate standard treatment, mainly due to severe side effects observed with use of these approaches. Moreover, the strategies that have had less adverse effects also did not demonstrate significant efficacy results. In parallel with these attempts, there is a continuous and growing understanding of signaling pathways involved in oncogenesis [72]. Under the context of developing therapies targeted, these signaling pathways, which are usually altered in specific tumor cells, provide new possible targets in hope of gaining some control over the malignant cells. 

Treatment of melanoma focusing on a molecular disease model: the basis of this principle is the recognition of melanoma as a heterogeneous disease and that tumorigenic cells are more variable at a molecular level than can be observed macro/microscopically [73]. Furthermore, the concept of “oncogene addition” describes that malignant cell depends more strongly on hyperactivated pathways than do normal cells, as well as the specific activated oncogenes that drive those pathways. Therefore, this therapeutic opportunity allows some control over the cancer cells through precise treatments according to genetic lesion that underlie each individual disease [2]. It is important to mention that not all oncogenes can be a treatable target. In general, powerful targets are enzymes such as kinases, proteases on account of their catalytic sites which assure satisfactory selectivity to designed drugs binding. Even now, there are countless possibilities for molecular targets. This number is so expressive that it was very recently proposed for formal process for classifying melanoma into molecular subtypes and developing proposed treatment guidelines for each subtype, including specific assays, drugs, and clinical trials. This “molecular disease model” can be used by clinicians to guide treatment decisions, and refined by researchers based on clinical outcomes and laboratory findings [73].  Table 5 summarizes the elegant initiative of Vidwans' research group who seek through this “dynamic” review article, a faster way to disseminate relevant new information and the continuous updating about this point. The table shows types and subtypes of melanoma in order of importance of the associated oncogene/tumor suppressor, prevalence, and potential for therapeutic intervention. According to Vidwans' research group, the principal subtypes (1.1–5.1) can act as a dominant oncogene and, therefore, are important foci for therapy while the subtypes described as secondary (6.1–8.1) play supportive role and generally coexist with mutations of the major subtypes. The latest version of the Melanoma Molecular Disease Model, besides other details, can be found online (available at http://mmdm.cancercommons.org/ml/index.php/A_Melanoma_Molecular_Disease_Model).

A large and permanently increasing number of pharmacological inhibitors targeting several of the recently identified mutated signal transduction molecules are being explored in various clinical trials as shown in Table 6. Most of the trials are still recruiting patients with advanced malignant melanoma who have the specific genetic profile to action of certain drugs. Although a few of these drugs have been approved for other types of cancer, as valproic acid, and sutent, dasatinib, some still have modest activity against melanoma, for example, sorafenib. As discussed before, molecular analyses of melanomas have revealed an activated *BRAF^V600E^* mutation as a promising biomarker to test the efficacy of BRAF-targeted therapy in treating malignant melanoma. Sorafenib monotherapy has shown to be inefficient because of its lack of specificity and low potency against the mutant *BRAF* [79, 80]. On the other hand, Flaherty and coauthors [24] conducted a multicenter, phase 1, dose-escalation clinical trial using PLX4032, a small molecule that acts selectively by targeting the mutation activated RAF/MEK/ERK signaling pathway. Afterwards, an extension phase was performed in order to identify the maximum dose that could be administered in phase 2 trials without significantly toxicity. As a result, the authors reported that treatment with PLX4032 at a dose of 960 mg orally twice daily induced responses in the majority of metastatic melanomas carrying *BRAF* mutations [24]. Importantly, Chapman and coworkers [80] are conducting a phase 3 interventional, randomized, controlled and multicenter clinical trial study (NCT01006980, Table 6) with the purpose of evaluating progression-free, overall survival, safety and tolerability of PLX4032 as compared with Dacarbazine in 675 metastatic melanoma patients previously untreated. As a result just yet, patients with unresectable stage IIIC or stage IV melanoma (AJCC) positive for *BRAF^V600E^* mutation receiving Vemurafenib (PLX4032, 960 mg orally twice daily) showed improved rates of overall and progression-free survival relative to patients who had received Dacarbazine (1000 mg/m^2^ of body surface area intravenously every 3 weeks) [80]. Taken together, these successes highlight the importance of a molecular disease model focusing on specific biomarkers, such as *BRAF^V600E^* mutation, as bona fide targets which could benefit melanoma patients. Another hypothesis to explain low activity of Sorafenib in melanoma cells is the possibility that melanoma cell proliferation could be driven by alternative pathways when RAF/MEK/ERK signaling is blocked [79, 81]. Based on this, several studies using inhibitors of distinct pathways have been initiated (Table 7) and encouraging response rates have been reported [82]. 

The amount of studies presented here and the emergence of new therapeutic opportunities show that the scientific community is really committed to finding the best way to overcome this challenge, the treatment for metastatic malignant melanoma. Since melanoma is much more than a single disease, the real benefit most likely will be require the use of multiple strategies together (chemotherapy, immunotherapy, targeted therapy). But if on one hand the positive synergistic effect of these agents is achieved by the other, an extra burden of toxicity and unknown events arising from the interaction of these agents cannot be ignored. It is known that, besides the intrinsic characteristics of each person, the biology of melanoma, as well as other types of cancer, has also a few distinct characteristics even among patients in same disease stage, which leads us to another challenge, before treatment choice: identifying the tumors and patients that best suited to respond to certain therapies.

## 6. Conclusion

Recent discoveries have provided fascinating insights into malignant melanoma development. It is clear that deregulation in distinct oncogenes and tumor suppressors are involved in malignant melanoma progression, as evaluated by results of compiled studies presented here. Thus, there are evidences in which more than one route leads to melanocyte full malignant transformation and melanoma progression. The major question to be considered by researchers, clinicians, and dermatopathologists is the limited success of discovered biomarkers to predict or identify groups of patients with substantial risk for metastasis. Unfortunately, no reliable biomarker that significantly translates into effectiveness therapeutic responses and overall median survival rate benefits to patients with advanced disease has been identified. Perhaps, this characteristic can be due to melanoma heterogeneity, substantial differences in experimental and clinical trials design, inadequate classification schemes, environmental-associated risk factors, and person's condition such as age, gender, and immune competence. Although a true biomarker is a molecule capable of distinguishing a specific stage of disease, the data reported here strongly suggest that understanding primary clonal events that occur in primary malignant melanomas through melanoma classification refinement may help us to identify molecular marks acting immediately downstream. These secondary alterations provide new insights for predicting the metastatic behavior in a more stable genomic, epigenomic, and metabolomic context. Hence, additional studies are required to identify distinct subpopulations of melanoma cells, including circulating tumor-initiating cells in attempt to identify these distinct molecular pathways and classify, in a more reliable way, patient candidates for further personalized medicine. Recently, Vidwans' group (2011) developed a molecular disease model that classifies melanomas into molecular subtypes based on genetic status of key biomarker(s)/pathway(s) and their combination, which are potential targets for existing therapeutic interventions [73]. This molecular classification of melanomas might be a powerful strategy used to refine melanoma classification and to guide appropriate target therapy. Thus, because melanoma is a heterogeneous disease, biomarkers can allow us to identify patients who best respond to certain therapeutic approach. And therefore standard treatments are currently available to be based primarily on usage, together or not, chemotherapies (dacarbazine) or/and imunotherapies (high-dose recommbinant IL-2, Ipilimumab), but require a rigorous preclinical evaluation as well as a constant medical supervision. On the other hand, another barrier for studying and treating melanoma metastasis is the limited availability of fresh melanoma tissues representing early stages of disease and adjacent normal skin. Because of this, different sources can be exploited to initial screening, such as an animal model that mimicries the “natural” evolution of disease. In this way, a murine melanoma progression model developed by our group [83] has shown good correlation with data reported in humans, especially with respect to epigenetic therapy, highlighting the potential translational application from the basic research to clinical research, which might be especially important in metastatic disease [16]. In conclusion, the characterization of distinct subpopulations of melanoma cells and signaling pathways by integrating classification scheme opens the avenue to the development of more responsive antimelanoma therapy.

## Figures and Tables

**Table 1:** Effects of candidates of oncogenic biomarkers in melanoma progression.

Biomarker	Biological effect(s) associated with biomarker upregulation	Reference
ABCB5	Associated with evasion of antitumor immunity and immunotherapeutic resistance	[31]

BPAG1	Detected as autoantibody in serum of melanoma patients	[32]

BRAF	Mediated melanoma cell resistance to *anoikis *	[33]

BRG1	Promoted epigenetic changes in extracellular matrix/adhesion gene expression and increased melanoma invasiveness	[34]

CD133 and nestin	Associated with poor prognosis	[35]

DEK	Associated with gain of chromosome 6p, proliferation, and chemoresistance	[36]

DNMT3B	Predicted overall survival in patients with lymph node metastases	[27]

HIF1A	Associated with a prosurvival role	[37]

Integrin *α*4*β*1	Required for lymphangiogenesis and metastasis	[38]

JARID1B	Required for continuous tumor growth	[21]

MCL-1	Required for melanoma cells resistance to *anoikis *	[39]

microRNA-200	Regulated morphological plasticity and determined modes of melanoma cells migration and invasion	[40]

MITF-Mdel	Purposed for diagnosis and followup	[41]

NG2/MPG	Correlated with multidrug resistance	[42]

S100A13	Represented a new angiogenic marker favoring the shift from radial to vertical tumor growth	[43]

TM9SF4	Associated with cannibalism behavior in metastatic lesions	[44]

TSPAN8	Mediated dermal invasion and progression to metastasis	[45]

Vimentin	Predicted hematogenous metastasis	[46]

WNT5A	Mediated melanoma metastasis	[47]

**Table 2:** Effects of candidate of tumor suppressor biomarkers in melanoma progression.

Biomarker	Biological effect(s) associated with biomarker downregulation	Reference
BAK	Weak BAK expression in serum of patients with melanoma was associated with lesions presenting “sparse” dermal nests	[48]

CIMP (WIF1, TFPI2, RASSF1A, and SOCS1)	Associated with advancing clinical stage of melanoma	[28]

KLOTHO	Associated with melanoma cell motility and invasiveness	[49]

microRNA-211	Associated with melanoma cells invasiveness	[50]

microRNA-29c	Predicted overall survival in patients with lymph node metastases	[27]

**Table 3:** Emerging immunotherapeutic to treatment of metastatic melanoma.

Target	Drug	Class	Phase(s) trial	Protocol_IDs*
CD40	CP-870,893	Fully human mAb	I	NCT01008527
			I	NCT01103635
CD137	BMS-663513	mAb	I	NCT00803374
Cytokines	Interleukin-21	Recombinant human molecule	II	NCT01152788
CTLA-4	Tremelimumab	Fully human IgG2 mAb	III	[62]
			II	NCT01034787
Immunocytokines	EMD 273063	Humanized anti-GD2 mAb linked to IL-2	II	NCT00590824
*α*_v_ integrin	CNTO95	mAb	I, II	NCT00246012
*α*_v_ *β*_3_ integrin	MEDI-522	Humanized mAb	I	NCT00111696
PD1	MDX-1106	Fully human mAb	I	NCT00730639
			I	NCT01024231
			I	NCT01176461
TGF-*β*	GC1008	Fully human mAb	I	NCT00899444
TGF-*β*2	AP12009	Antisense oligonucleotide	I	NCT00844064
TLR regulation of T_reg_ cells	CpG 7909 (ProMune)	Synthetic oligonucleotide	II	NCT01266603
			No phase specified	NCT00471471

*Randomized clinical trials selected from http://www.clinicaltrials.gov/.

**Table 4:** Recent report vaccine approaches to advanced malignant melanoma.

Phase trial	Purpose and brief comments	Reference
II	Treatment of 54 patients with a patient-specific tumor cell vaccines consisting of autologous dendritic cells, incubated with IL-4 and suspended in GM-CSF, which had phagocytized irradiated tumor cells from an autologous tumor cell line. Treatment was well-tolerated and the projected 5-year survival rate is an impressive 54% at a median followup of 4.5 years for the 30 surviving patients.	[74]

II	Immunization of unresectable stage III or stage IV M1a melanoma patients with recombinant MAGE-A3 protein combined with adjuvant systems AS15 or AS02B. The combination of MAGE-A3 and AS15 yielded higher specific Ab titers, more robust T-cell induction and long-lasting clinical responses or SD in metastatic melanoma.	[75]

II	To identify markers predictive of the clinical activity of the MAGE-A3 antigen-specific cancer immunotherapeutic (ASCI) from gene expression profiling by microarrays. A gene-signature derived from pretreatment tumor biopsies has been developed and shown to predict clinical benefit.	[76]

II	Routinely intratumoral injections of OncoVEX^GM-CSF^, an oncolytic herpes simplex virus vector encoding granulocyte monocyte colony-stimulating factor (GM-CSF). It was observed an improvement rate and durability of response when compared to other treatment options available to patients with advanced melanoma.	[77]

III	Learn more about the safety and risks of using OncoVEX^GM-CSF^. Results of a phase II trial of OncoVEX^GM-CSF^ were encouraging and led to the design of this Phase III trial.	NCT00769704*

III	Comparative study between metastatic melanoma patients treated with gp100: 209–217(210M) peptide followed by high-dose IL-2 and high-dose IL-2 alone. The peptide vaccine plus HD IL-2 promoted significant improvement in progression-free survival (PFS) without a clear impact on survival.	[78]

*Clinical trials selected from http://www.clinicaltrials.gov/.

**Table 5:** Principal and secundary melanoma molecular subtypes [73].

Detailed subtypes	Pathway(s)	Key gene/biomarker(s)	Potentially relevant therapeutics
1.1	MAPK	BRAF	BRAF inhibitors, MEK inhibitors, Hsp90 inhibitors
1.2	MAPK	BRAF/PTEN	(BRAF inhibitors) AND (PI3K inhibitors, AKT inhibitors or mTOR inhibitors)
1.3	MAPK	BRAF/AKT	(BRAF inhibitors) AND (AKT inhibitors or mTOR inhibitors)
1.4		BRAF/CDK4	BRAF inhibitors AND CDK inhibitors
2.1	c-KIT	c-KIT	Gleevec & other c-KIT inhibitors
3.1	GNAQ GNA11	GNAQ	MEK inhibitors
3.2	GNAQ GNA11	GNA11	MEK inhibitors
4.1	NRAS	NRAS	MAPK & PI3K inhibitors, Farnesyl transferase inhibitors
5.1	MITF	MITF	HDAC inhibitors
6.1	AKT/PI3K	PTEN	PI3K inhibitors, AKT inhibitors or mTOR inhibitors
6.2	AKT/PI3K	AKT	AKT inhibitors or mTOR inhibitors
6.3	AKT/PI3K	PI3K	PI3K inhibitors, AKT inhibitors or mTOR inhibitors
7.1	CDK	ARF/INK4	CDK inhibitors
7.2	CDK	CDK4	CDK inhibitors
7.3	CDK	CCND1/Cyclin D1	CDK inhibitors
8.1	PR3/BCL	BCL-2	TBD
8.2	PR3/BCL	P53	TBD

9	Placeholder for any new subtype of patients that is not currently defined		

**Table 6:** Selected drugs for targeted melanoma therapy.

Target(s)	Drug	Phase(s) trial	Protocol_IDs*
BRAF	PLX4032	II	NCT00949702
		III	NCT01006980

ARAF	RAF265	In Phase 1 for malignant melanoma	NCT00304525
BRAF			
CRAF			
VEGFR-2			

RAF PDGFR VEGFR-2	Sorafenib	Modest activity against melanoma	—

BRAF	GSK2118436	III	NCT01227889
		II	NCT01153763

MEK	AZD6244	II	NCT00866177

MEK	GSK1120212	III	NCT01245062

MEK	AZD8330	In phase I for (advanced malignancies)	NCT00454090

Hsp90	AT13387	In phase I for (solid tumors)	NCT00878423

c-KIT	Dasatinib	II	NCT00436605

c-KIT	Gleevec	III,	NCT00470470
		II	NCT00667953

c-KIT	Tasigna	III	NCT01028222

c-KIT	Sutent	II	NCT00631618

GNAQ	GSK1120212	III	NCT01245062
GNA11			

HDAC	Panobinostat	I,	NCT00925132
		III	NCT01065467

HDAC1	Valproic acid	I	NCT00495872

PI3K			
MTOR1	SF1126	In phase I for (advanced malignancies)	NCT00658671
MTOR2			

AKT	MK2206	In phase I for (solid tumors)	NCT00848718

MTOR	OSI-027	In phase I for (advanced solid tumors)	NCT00698243

CDK4	Flavopiridol/alvocidib/HMR 1275	II	NCT00005971

CDK4	P276-00	II	NCT00835419

CDK4	UCN-01	II	NCT00072189

*Randomized clinical trials selected from http://mmdm.cancercommons.org/ml/index.php/A_Melanoma_Molecular_Disease_Model.

**Table 7:** Dual Therapeutic intervention.

Targets	Drug	Phase(s) trial	Protocol_IDs*
BRAF and MEK for the MAPK pathway	Sorafenib and Temsirolimus	I, II	NCT00349206
PI3K, AKT and mTOR for the AKT/PI3K pathway	MK2206 plus AZD6244	I	NCT01021748

*Clinical trials selected from http://mmdm.cancercommons.org/ml/index.php/A_Melanoma_Molecular_Disease_Model.
